# The Role of Green Tea Catechin Epigallocatechin Gallate (EGCG) and Mammalian Target of Rapamycin (mTOR) Inhibitor PP242 (Torkinib) in the Treatment of Spinal Cord Injury

**DOI:** 10.3390/antiox12020363

**Published:** 2023-02-03

**Authors:** Lucia Machova Urdzikova, Veronika Cimermanova, Kristyna Karova, Jose Dominguez, Katerina Stepankova, Michaela Petrovicova, Katerina Havelikova, Chirag D. Gandhi, Meena Jhanwar-Uniyal, Pavla Jendelova

**Affiliations:** 1 Institute of Experimental Medicine, Czech Academy of Sciences, Vídeňská, 1083 Prague, Czech Republic; 2 Departments of Neurosurgery, New York Medical College, Valhalla, NY 10595, USA; 3 Department of Neuroscience, Second Faculty of Medicine, Charles University, V Uvalu 84, 150 06 Prague, Czech Republic

**Keywords:** spinal cord injury, mTOR pathway, EGCG, PP 242, inflammatory response, neuroregeneration, astrogliosis, axonal growth

## Abstract

Spinal cord injury (SCI) is a devastating condition that has physical and psychological consequences for patients. SCI is accompanied by scar formation and systemic inflammatory response leading to an intense degree of functional loss. The catechin, epigallocatechin gallate (EGCG), an active compound found in green tea, holds neuroprotective features and is known for its anti-inflammatory potential. The mammalian target of rapamycin (mTOR) is a serine/threonine kinase that exists in two functionally distinct complexes termed mTOR complex 1 and 2 (mTORC1; mTORC2). Inhibition of mTORC1 by rapamycin causes neuroprotection, leading to partial recovery from SCI. In this study the effects of EGCG, PP242 (an inhibitor of both complexes of mTOR), and a combination of EGCG and PP242 in SCI have been examined. It has been found that both EGCG and PP242 significantly improved sensory/motor functions following SCI. However, EGCG appeared to be more effective (BBB motor test, from 2 to 8 weeks after SCI, *p* = 0.019, *p* = 0.007, *p* = 0.006, *p* = 0.006, *p* = 0.05, *p* = 0.006, and *p* = 0.003, respectively). The only exception was the Von Frey test, where EGCG was ineffective, while mTOR inhibition by PP242, as well as PP242 in combination with EGCG, significantly reduced withdrawal latency starting from week three (combinatorial therapy (EGCG + PP242) vs. control at 3, 5, and 7 weeks, *p* = 0.011, *p* = 0.007, and *p* = 0.05, respectively). It has been found that EGCG was as effective as PP242 in suppressing mTOR signaling pathways, as evidenced by a reduction in phosphorylated S6 expression (PP242 (*t*-test, *p* < 0.0001) or EGCG (*t*-test, *p* = 0.0002)). These results demonstrate that EGCG and PP242 effectively suppress mTOR pathways, resulting in recovery from SCI in rats, and that EGCG acts via suppressing mTOR pathways.

## 1. Introduction

Spinal cord injury (SCI) can lead to neuronal dysfunction, which is the major cause of disability in adults. The biphasic nature of SCI consists of primary and secondary injuries. Primary injury is the mechanical damage to the spinal cord. In this phase of injury, there is loss of synaptic connections, damage to demyelination and axons, and mechanically induced cell death of neurons. The secondary injury that follows the primary injury is more deleterious and involves a cascade of inflammatory, vascular, and biochemical events that further impair neuronal function. These primary and secondary injury events activate fibroblasts and glia, including astrocytes, pericytes, Schwann cells, and microglia [1]. The dialog between activated glia and injured neurons underlies endogenous pathological and reparative processes in the injured central nervous system (CNS). Traumatic SCI is characterized as a permanent or temporary loss of function of the spinal cord that is usually caused by physical impact on the spine that dislocates or fractures vertebrae [2,3]. This sub-acute phase includes mechanisms such as oxidative stress, mitochondrial dysfunction, decreased ATP production, activation of astrocytes and microglia, immune cell invasion, the release of cytokines, mediated cell death, and others [4]. While primary injury is irreversible, the secondary injury processes are a target of many experimental therapeutic interventions as they can be, to some extent, reversible. Several natural polyphenolic compounds with free radical scavenging, anti-inflammatory, and anti-apoptotic properties have recently been shown to have therapeutic values. The chemical composition of green tea comprises polyphenols, known as catechins [5]. Epigallocatechin gallate (EGCG) is the most abundant catechin compound found in green tea, which has been shown to be responsible for its biological and beneficial activity [6], and is also known for its anti-inflammatory potential and neuroprotective properties [7]. We reported that EGCG decreases the nuclear translocation of subunit p65 (RelA) of the NF-κB dimer, therefore attenuating the canonical NF-κB pathway [8]. In several studies, EGCG administration after neural damage led to a reduction of neuropathic pain [9,10]. EGCG–selenium nanoparticles, when delivered intravenously to rats, significantly improved locomotor functions by protecting neurons and myelin sheets due to the ROS-scavenging and anti-inflammatory activity [11]. Oral administration of EGCG enhanced pain management of patients with epidural analgesia and multiple rib fractures [12]. The role of EGCG was tested in human clinical studies in cancer prevention [13], the treatment of multiple sclerosis [14,15], and cardiac and metabolic health [16]. Curcumin [17,18,19] and EGCG [20,21,22] are used for their anti-oxidative, chelating, and immunomodulating properties in various diseases. In these therapies, TNF-α and IL-1 level reduction is one of the key factors in their pro-regenerative features after SCI [7,23], and may result in a synergistic effect of curcumin and EGCG [24]. To date, a combination of both drugs is used as a complementary therapy in experimental cancer therapy [25,26]. EGCG treatment has led to an increase in the survival of neurons, as well as higher GAP-43 immunoreactivity in chronic SCI associated with improved locomotor functions [27]. Furthermore, several studies have shown the effect of EGCG on edema, tissue protection, and functional recovery after SCI [28,29]. Despite that, all the mechanisms of action of these compounds are unknown.

The mammalian target of rapamycin (mTOR) is a serine/threonine protein kinase that plays a role in the regulation of cell metabolism, proliferation, cell death, and survival. mTOR is involved in transcription and translation, vesicular trafficking, cytoskeletal organization, and also plays a role in autophagy. The mTOR pathway is one of the most studied signaling pathways and is affected in many pathologies, such as Parkinson disease, Alzheimer disease, and brain and spinal cord trauma. It is well known that the inhibition of mTOR can reduce neural tissue damage in CNS injuries [30,31]. mTOR regulates axonal regeneration after SCI [32,33] and reduction of astrocytic scar formation in the injured spinal cord. Inhibition of mTOR reduces neural tissue damage and locomotor impairment after spinal cord injury [34]. The inhibition of the mTOR signaling pathway has been suggested as a promising pharmacological option to tackle spinal cord injury due to the regulation of neuronal cell growth, survival, axonal and dendritic development during differentiation, and synaptic plasticity [35,36]. mTOR is a core component of the signaling network, which belongs to the phosphoinositide 3-kinase- (PI3K-) related protein kinase family. It assembles into two complexes, mTORC1 and mTORC2, which are composed of subunits raptor and rictor, respectively. The activation of the PI3K/Akt/mTOR signaling pathway involves glial scar formation. The inhibition of this pathway by rapamycin-reduced macrophage/neutrophil infiltration into the lesion site, microglia activation, and secretion of TNF-α, inhibited astrocyte proliferation at the lesion site and increased neuronal survival and axonogenesis towards the lesion site [37,38]. PP242 is a second generation mTOR inhibitor that suppresses signaling pathways mediated by mTORC1 and mTORC2. Treatment with PP242 caused distinct inhibition of the mTOR pathway in the spinal cord tissue after SCI. PP242 reduced lesion size after SCI, improved axonal growth, and also improved motor abilities in rats 7 days after induction of the lesion [38]. In addition, as a dual inhibitor of the mTOR pathway, PP242 potentiated the autophagic response during treatment compared to the mTORC1 specific inhibtor rapamycin. Also, PP242 affected systemic inflammatory reactions [38].

Polyphenols are known for their capacity to manipulate mTOR pathways. EGCG reduces the expression of both p85 (the regulatory subunit of PI3K) and phosphorylated Akt (Ser473) [39]. Van Aller [40] showed that EGCG and related catechins are inhibitors of all four class I PI3K isoforms (PI3Kα, PI3Kβ, PI3Kγ, and PI3Kδ). In our study, the effects of two mTOR inhibitors, PP242 and a green tea polyphenol EGCG, on the development of the spinal cord lesion and functional motor and sensory recovery have been compared. The present study was performed to understand how both components influence via modulation of mTOR pathway development of spinal cord lesion, and also how the treatment with both components influences secondary processes after SCI. The present study may contribute to the development of a novel strategy for the treatment of spinal cord injury.

## 2. Materials and Methods

### 2.1. Animals

Male Wistar rats, 10 weeks old, (*n* = 71; Breeding facility of the Physiological institute CAS, Prague, Czech Republic), weighing approximately 300 ± 15 g, were used in this study. Rats were housed in pairs in individually ventilated cage systems (Tecniplast, Buguggiate, Italy), and provided with food and water *ad libitum*. Immediately following SCI, all animals were randomly divided into 4 subgroups for behavioral examinations: control (*n* = 13), PP242 (*n* = 13), EGCG (*n* = 11), and PP242 + EGCG (*n* = 10). PP242 (5 mg/kg; Apex BIO, Houston, MA, USA) [38], EGCG (50 mg/kg; Merck, Branchburg, NJ, USA) [8], combination of PP242 (5 mg/kg) + EGCG (50 mg/kg,), or vehicle in controls was administered intraperitoneally. The rats received treatment daily for 5 consecutive days, starting from the second day after SCI. The rats (*n* = 47) survived for 9 weeks after SCI surgery. The rats were behaviorally tested according to the protocol and their spinal cord tissue was removed for histological evaluation of white and gray matter, astrogliosis, and axonal sprouting. In addition, immunohistological analysis for mTOR signaling molecule pS6 and PCR analysis for cytokines were also performed. A total of 24 rats survived for 7 days after SCI, and their spinal cords were used for immunohistochemistry (*n* = 6, each group). The number of animals were estimated according to the power analysis based on the data from our previous experiments. All the experiments were performed in accordance with the European Communities Council Directive of 22 September 2010 (2010/63/EU) regarding the use of animals in research and approved by the Ethics Committee of the Institute of Experimental Medicine CAS and subsequently by the Section Committee of Czech Academy of Sciences, Prague, Czech Republic (Project No. 54/2017, approved 14 July 2017). The number of animals was statistically optimized to achieve refinement and reduction.

### 2.2. Spinal Cord Injury

Spinal cord injury was induced by the balloon-compression model. According to Vanicky et al. [41], the rat’s rectal temperature was kept at 37 °C with a heating pad throughout the surgery to avoid hyperthermia [42]. After induction of anesthesia, dorsal laminectomy on the T10 vertebrae enabled the insertion of a 2 French Fogarty catheter (Edwards Lifesciences, Irvine, CA, USA) into the epidural space and the positioning of the center of the balloon at T8 spinal level. Subsequently, the balloon was inflated with 15 μL of saline solution for 5 min. Afterwards, the balloon was removed, and the soft tissue and skin were closed in anatomical layers. The surgery procedure was completed by administering antibiotics (ampicillin 60 mg/kg, onceper day, for 5 days, Biotika, Prague, Czech Republic) and buprenorphine (Vetergesic Multidose for cats and dogs 0.05–0.1 mg/kg, as needed, Ceva Santé Animale, Libourne, France) to prevent infection and subsequent pain. The complete paraplegia was developed in rats and was followed by progressive recovery within 8 weeks. During the recovery phase, the animals were assisted with feeding and manual bladder expression due to urine retention. Throughout the whole experiment, the rats had access to water, standard rat chow *ad libitum* and were kept at a 12 h light/dark cycle. The health conditions of the animals were examined daily for potential postoperative SCI complications.

### 2.3. Functional Analysis

#### 2.3.1. BBB Test

Basic locomotor function was assessed by BBB open-field locomotor test [43]. Rats were placed in an open field area for approximately 4-min/week starting 7 days post SCI. The scoring was done by two independent examiners according to the BBB scale (0–21). The grading was determined as a scale which was given as follows: scale 0–4 points reflect no or only minor hindlimb movement with no weight support; scale 5–8 points show a larger extent of movement with no weight support; scale 9–14 points suggest increasing frequency of weight-supported steps and hindlimb coordination; scale 15–18 points reflect precise movements such as specific paw placement rotation; scale 18–20 points indicate tail balance and trunk stability; and scale 21 points indicate healthy animals. Two rats were placed into the round open field arena for 4 min. Both hindlimbs of the animal were scored separately. The final BBB score for each rat is the average from the BBB score of the right and left hindlimbs.

#### 2.3.2. Flat Beam Test

To evaluate the advanced locomotor skills of injured rats, such as weight support and hindlimb coordination, the flat beam test was performed [44]. The latency (in seconds) and trajectory of rats crossing the beam were recorded for evaluation. The scoring was done according to the modified version of the Goldstein scale (0–7) [44]. The flat beam test was designed to measure the ability to maintain balance on a beam (scale points 0–2), attempts to cross the beam (scale points 2–3), the frequency of hindlimbs use, hindlimb coordination technique, and gait (scale points 4–7). The time score reflects the time that each rat needs to start performing the task (maximum is 60 s). Following a pre-training session, rats were assessed twice a day for 3 consecutive days, and then weekly starting the third week after SCI.

#### 2.3.3. Von Frey Test

The Von Frey test was used to determine sensitivity to a mechanical nociception [45]. The Von Frey apparatus (IITC IncLife Science, Woodland Hills, CA, USA) is composed of transparent boxes with a mesh platform and Von Frey fibers (a pipette tip was used). Rats were placed into the transparent box and allowed to acclimatize for 15 min. The Von Frey rigid tip pressure was slowly increased against the hind paw until a nociceptive withdrawal response was received. The value was recorded and measurements repeated until 5 values were measured for each hind paw. The average of 5 values was used for statistical evaluation.

#### 2.3.4. Ladder Walking Test

The Ladder walking test was performed on the MotoRater unit TSE Systems (TSE-Systems Inc, Bad Homburg, Germany) to evaluate qualitative and quantitative motor functions [45]. The instrument consists of Plexiglas walls, side mirrors, a bottom camera, and a goal black box (a motivation to hide). Metal rungs (horizontal ladder) were added to the MotoRater unit for the Ladder walking test. Rats were placed at the start of the ladder and motivated to cross the ladder to the terminal black box, while being captured by the bottom camera. Recorded videos were evaluated by a qualitative evaluation of hindlimb placement 8 weeks after SCI (foot placement accuracy analysis) or by the number of error placements in the passage 4 weeks after SCI (foot fault scoring). Each rat was recorded 4 times and each hind paw was analyzed separately.

#### 2.3.5. Testing Schedule

Rats were pre-trained a week before spinal cord injury for the Beam walk test, Von Frey test, and the Ladder test. The BBB test was performed weekly after SCI. The Beam walk test was executed twice a week from the fourth week after SCI. Von Frey tests were carried out alternately, every other week after SCI, starting with the Von Frey test. The Ladder Foot fault scoring was executed in the fourth week after SCI and the Ladder Foot placement accuracy analysis was recorded in the eight week after SCI.

### 2.4. Histological and Immunohistochemical Analysis

To analyze the effect of EGCG, PP242, or the combined therapy on injured spinal cords, 15 cross-sections (5 μm thickness) per spinal cord were selected at 1-mm intervals along the cranio-caudal axis, including the lesion center. Cresyl violet-Luxol fast blue staining was performed to distinguish the white and gray matter (WM/GM) of the injured spinal cord. The total area and distribution of sparse white/gray matter and cavity size were analyzed using ImageJ software (NIH, Bethesda, MD, USA) [41].

All 71 rats were used for histological and immunohistochemical analyses. A total of 47 animals underwent transcardial perfusion with 4% paraformaldehyde in the eighth week after SCI, and 24 animals were transcardially perfused 7 days after SCI. Concisely, anesthetic pentobarbital (150 mg/kg. Penbital, Bioveta, Ivanovice na Hane, Czech Republic) was injected intraperitoneally to overdose the animals. After thoracotomy, transcardial perfusion was started with a phosphate buffer followed by 4% paraformaldehyde in the phosphate buffer. Rat’s spines were removed and placed in 4% paraformaldehyde for additional overnight fixation. The following day, spinal cords were dissected (2 cm superior and 2 cm inferior from the lesion) and left in 4% paraformaldehyde for the following procedures.

Paraffin-embedded and serially sectioned spinal cords (5 μm cross-sections, 1 mm interval) were used for further morphometric and immunohistochemical analyses. Luxol Fast Blue histological staining was chosen for sparing areas of white and gray matter analysis. Axioskop 2 plus microscope (Zeiss, Oberkochen, Germany) and Image J software (Wayne Rasband, NIH, Bethesda, MD, USA) were used for capturing and analyzing images [41].

A series of spinal cords were immunostained for detection of astrogliosis and axonal sprouting. Immunostaining for astrogliosis was performed using primary anti-GFAP antibody conjugated to CY3 (MAB3402C3, 1:200, Sigma, St, Louis, MO, USA). Axonal sprouting was detected by a primary antibody against GAP-43 (AB5220, 1;1000 Millipore, Billerica, MA, USA) and a secondary antibody goat anti-mouse IgG conjugated to Alexa-Fluor 488 (ab150113, 1:500, Abcam, Bristol, UK). Images were captured with a LEICA CTR6500 with FAXS 4.2.6245.1020 software (TissueGnostics, Vienna, Austria). For immunohistochemistry, images were analyzed using Image J software (Wayne Rasband, NIH, Bethesda, MD, USA) for astrogliosis and HistoQuest 4.0.4.0154 software (TissueGnostics, Vienna, Austria) for axonal sprouting [8].

### 2.5. Estimation Using Quantitative Reverse Transcription-PCR (qRT-PCR)

Expression changes in the transcription of rat target genes Vegfa, Fgf2, Cntf, and Gap43 9 weeks after SCI with *n* = 4–6 per group were measured using a quantitative real-time reverse transcription polymerase chain reaction (qRT-PCR). RNA was isolated from paraformaldehyde-fixed spinal cord tissue sections using the High Pure RNA Paraffin Kit (Roche, Penzberg, Germany). Isolated RNA was quantified with spectrophotometer (NanoPhotometerTM P-Class, Munchen, Germany), then reverse transcribed into cDNA with Transcriptor Universal cDNA Master (Roche, Penzberg, Germany) and a thermal cycler (T100™ Thermal Cycler, Bio-Rad, Hercules, CA, USA). All reactions were performed using cDNA solution, FastStart Universal Probe Master (Roche, Penzberg, Germany), and TaqMan^®^ Gene Expression Assays (Life Technologies, Carlsbad, CA, USA): glyceraldehyde 3-phosphate dehydrogenase/Gapdh/Rn01775763_g1, vascular endothelial growth factor A/Vegfa/Rn01511601_m1, basic fibroblast growth factor/Fgf2/Rn00570809_m1, ciliary neurotrophic factor/Cntf/ Rn00755092_m1, and growth associated protein 43/Gap43/Rn01474579_m1. The final reaction volume was 10 μL containing 20 ng of extracted and reverse transcribed RNA. A real-time PCR cycler (StepOnePlus™, Life Technologies, Carlsbad, CA, USA) was used for amplification with the following cycling conditions: 2 min at 50 °C, 10 min at 95 °C, followed by 40 cycles of 15 s at 95 °C and 1 min at 60 °C. Relative quantification of gene expression was determined using the ΔΔCt method. The data was analyzed with StepOnePlus^®^ software (Life Technologies, Carlsbad, CA, USA). For normalization of gene expression levels, Gapdh was used as a reference gene. A log2 scale was used to display the magnitude for up- and down-regulated genes, as values from treatment groups were plotted against the SCI-only group. Statistical analysis was performed from ΔCt values of controls as well as treated animals [8].

### 2.6. Statistical Analysis

All the raw data from behavioral testing, histological, and immunohistochemical parts of the study were summarized in Excel (Office 2010, Microsoft). The statistical comparison of the four tested groups (EGCG, PP242, combination of the two, and vehicle-treated tested group) was accomplished with several statistical tests in the GraphPad Prism for Windows software (version 5.03, GraphPad Software, Boston, MA, USA). For evaluation of the normality of the raw data, Kolmogorov–Smirnov test was used. For behavioral testing, the two-way repeated measurement ANOVA was applied, except for the Ladder walking test, where the one-way ANOVA was used. Immunohistochemical GFAP analysis was analyzed with the two-way ANOVA mixed-effects model (REML). The Bonferroni correction test was used as a post hoc pair-to-pair test. The tests were selected according to the distribution of the data for each investigation.

All graphs in the results part display as arithmetical means with standard error of the mean. The statistical significance in the graphs as well as in the text is signposted by a scheme; * *p* < 0.05, ** *p* < 0.01, *** *p* < 0.001.

## 3. Results

### 3.1. Behavioral Performance of the Rats after Vehicle, EGCG, PP242, and EGCG + PP242 Treatment

#### 3.1.1. BBB Test

**Figure 1:**
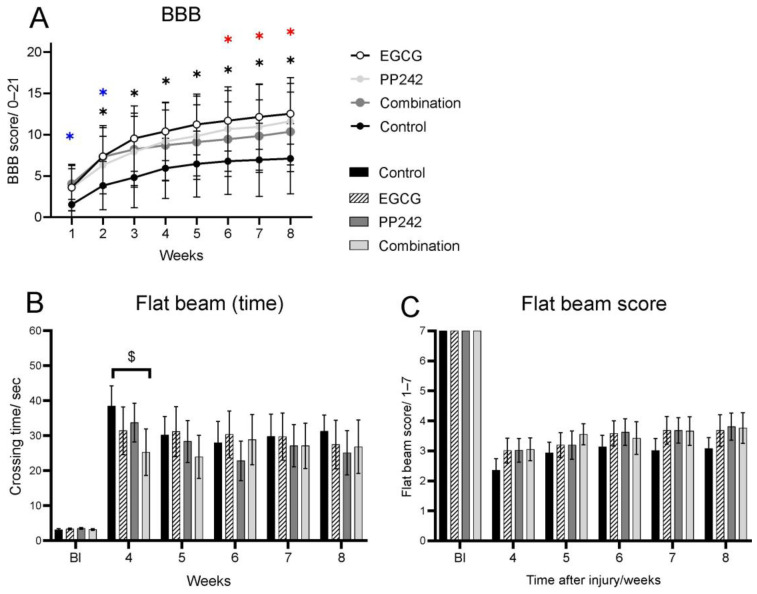
BBB and flat beam test in rats treated with vehicle, EGCG, PP242, and EGCG + PP242 (**A**) BBB test shows improvement of the motor performance score the first and second week after SCI, when compared to EGCG + PP242 and the vehicle-treated group. From the second week to the end of the survival time, the EGCG=only treatment significantly improved the motor performance of the rats compared to controls. The PP242 treatment also led to significant improvement compared to the controls 6, 7, and 8 weeks after SCI. (**B**,**C**). Flat beam test score showed no significant difference among any treated groups including controls; however, the time to cross the beam was significantly shorter in EGCG + PP242-treated rats in week two. * EGCG vs. Control, * PP242 vs. Control. * Combination vs. Control, (* *p* < 0.05), ($ *p* 0.05–0.1).

Behavioral recovery following SCI was evaluated by the BBB of hindlimb open-field locomotor test. The day after SCI, rats were paraplegic, starting with a score from 0 to 1. However, all four groups displayed gradual recovery from the first week on, and two-way ANOVA rendered a significant treatment and time response (Two-way RM ANOVA, F = 2.085, *p* = 0.0040). Rats starting with a higher score were excluded from the study. As shown in Figure 1A, behavioral recovery of rats treated with saline reached a plateau 4 weeks after injury, with an average BBB score of less than 6. However, EGCG-treated rats showed a significant improvement in locomotor recovery as observed by higher BBB. The rats treated with EGCG reached a significantly higher BBB score from 2–8 weeks after SCI (*p* = 0.019, *p* = 0.007, *p* = 0.006, *p* = 0.006, *p* = 0.05, *p* = 0.006, and *p* = 0.003, respectively). Treatment with mTOR inhibitor PP242 also displayed a significantly improved BBB score over the course of 6, 7, and 8 weeks (*p* = 0.042, *p* = 0.047, and *p* = 0.025), and there was a strong trend (*p* = 0.05–0.1) from 1–5 weeks after SCI. However, this was less effective than the EGCG-treated group. Combined treatment with EGCG + PP242 caused a significantly higher recovery in comparison with saline-treated rats. However, it was less effective than EGCG treatment alone. As shown in Figure 1A, in the first and second week after SCI, the rats treated with a combination of EGCG and PP242 reached a significantly higher BBB score compared to the vehicle-treated group (*p* = 0.004 and *p* = 0.022, respectively) and a strong trend towards significance (*p* = 0.061) was seen in the third week after SCI. Saline-treated rats also showed consistently improved BBB scores over the course of 8 weeks, but remained noticeably lower than treated groups at all time points.

#### 3.1.2. Flat Beam Test and Flat Beam Score

Advanced locomotor and coordination of the hindlimb skills after SCI were evaluated by the flat beam test. In the flat beam test, a physically demanding test, saline-treated animals reached an average value of 1, which represents the ability to balance on the beam for at least 30 s consecutively without being able to cross it. The walking performance on the flat beam expresses very well the fine motor coordination and body balance of the rats after SCI. EGCG-, PP242-and-EGCG-, and PP242-treated rats showed higher scores when compared to the saline-treated controls, although they failed to reach significance (ANOVA F = 0.5120, *p* = 0.9326). Similarly, time to cross the beam was significantly lower in all treated groups with statistically significant change in the combined group in week four. However, there were no significantly different results between all four groups’ flat beam time scores (ANOVA, time: F = 0.6459, *p* = 0.8346; Figure 1B,C).

#### 3.1.3. Ladder Walking Test

**Figure 2:**
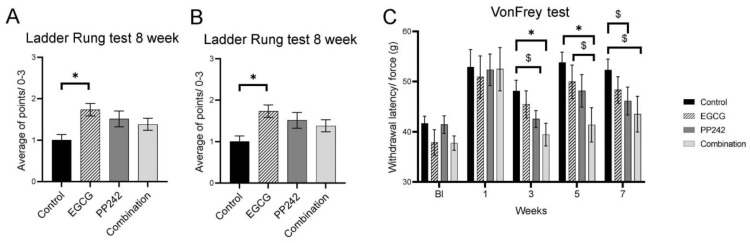
Motor performance test, ladder rung test, and mechanical sensitivity test by Von Frey test. (**A**,**B**) The complex motor performance test using ladder rung test revealed no performance difference among all tested groups in the fourth week; at 8 weeks after SCI, EGCG treatment significantly improved motor performance in the ladder rung test. (**C**) Mechanical sensitivity using the Von Frey test displayed significantly better withdrawal latency at weeks three and five after SCI in EGCG + PP242-treated rats (* *p* < 0.05), ($ *p* 0.05–0.1).

The Ladder walking test represents a complex motor performance test that includes trunk stability, coordination, and placement of the paw while walking. The ability to walk on the ladder was improved during the survival time, as shown in Figure 2A. As the rats were unable to place their paws properly on the rungs 4 weeks after SCI, incorrect steps were recorded and evaluated as the ratio of all steps taken relative to incorrect steps. There was no statistical significance between groups in week four (One-way ANOVA test, F = 1.083, *p* = 0.3666). A significant improvement in walking performance was seen in week eight. As the motor performance was improved (seen also on BBB test (Figure 1A), the rats were able to place their paws on the rungs better, and their score was evaluated on a scale from 0 to 3. By week eight, rats treated with EGCG reached a significantly better ladder walking score than the control group (*p* = 0.0137) (Figure 2B). (One-way ANOVA test, F = 3.796, *p* = 0.0168). PP242 and combined treatment with EGCG + PP242 also improved in performance in ladder walking. However, they did not reach statistical significance.

#### 3.1.4. Von Frey Test

Sensitivity to mechanical stimulus was evaluated by the Von Frey test. When comparing pre-injury values with those in the first week after SCI, the response to mechanical stimulation was prolonged. In the combined treatment group (EGCG + PP242), the sensitivity was restored almost to the pre-injury level. A significantly shorter response time to mechanical stimulation in rats treated with combinatorial therapy (EGCG + PP242) at 3, 5 and 7 weeks (*p* = 0.011, *p* = 0.007, and *p* = 0.05, respectively) was observed. A significantly shorter response time was also seen in PP242 in week three compared with controls (*p* = 0.05), and a trend towards significance was also seen when compared with controls in week seven after SCI (*p* = 0.098)(Two-way RM ANOVA, F = 0,7677, *p* = 0.6830).

### 3.2. Histopathology

#### 3.2.1. White and Gray Matter Sparing

**Figure 3:**
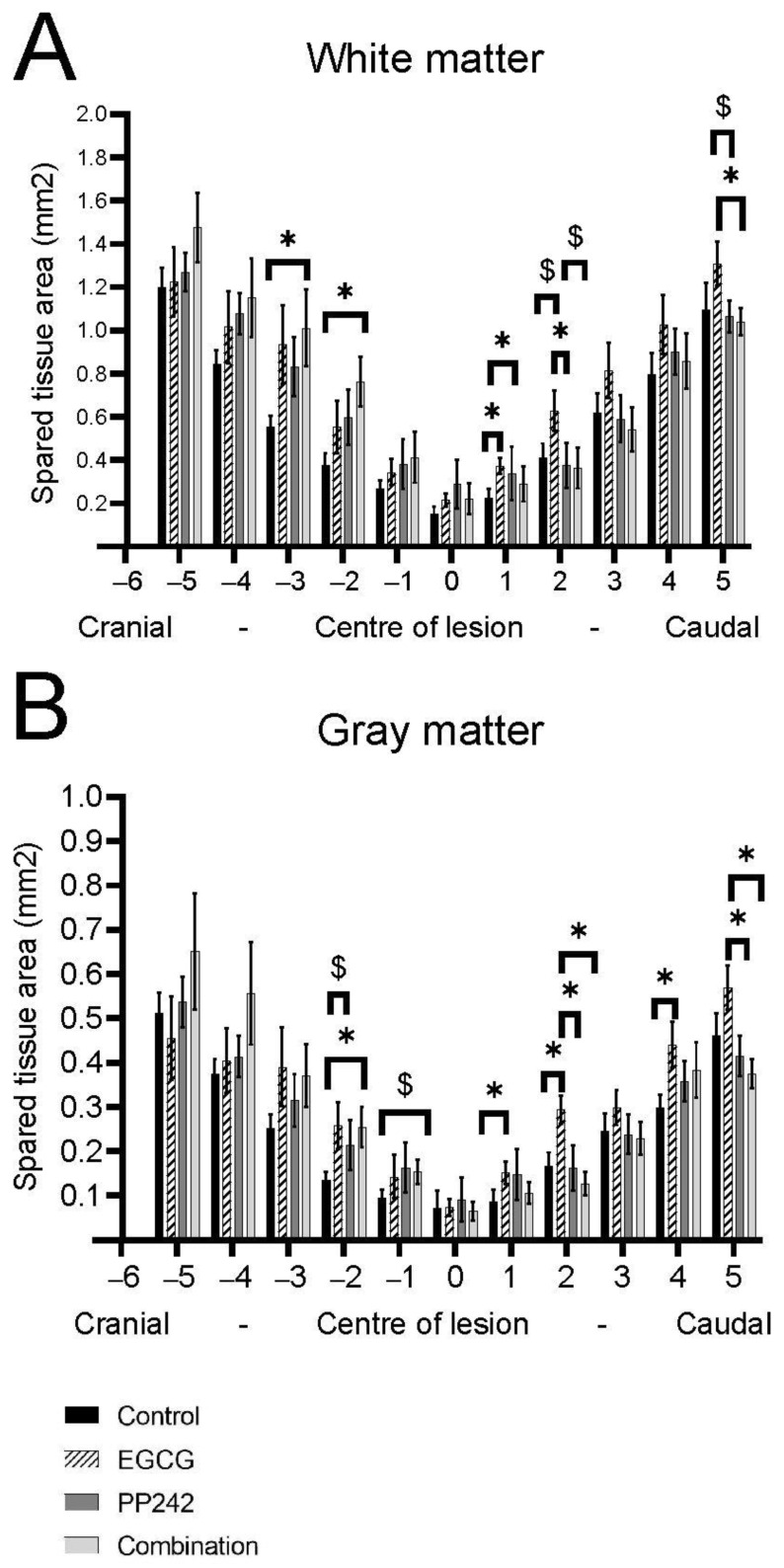
Histopathological and immunohistochemical investigation of the spinal cord lesion. The analysis of the remaining white and gray matter displayed higher preservation of the white and gray matter (**A**) after EGCG treatment compared to other treated groups and controls. The combinatory treatment was also efficient in protecting white and gray matter compared to controls (**B**) (* *p* < 0.05), ($ *p* 0.05–0.1).

Luxol Fast Blue staining was used to distinguish white and gray matter (Figure 3). The area of remaining white matter was measured along every millimeter of the spinal cord lesion on five sections cranially and five sections caudally from the lesion center 8 weeks after SCI. EGCG treatment alone had a protective effect on white matter, displaying extra preservation of WM tissue compared to control (1 mm, *p* = 0.013), PP242 (1 mm, *p* = 0.016; 2 mm, *p* = 0.01), and combinatory treatment (5 mm, *p* =0.038). The treatment with EGCG + PP242 also resulted in greater preservation of the white matter tissue cranial to the center of the lesion (−2 mm, *p* = 0.16; −3 mm, *p* = 0.032) (Two-way RM ANOVA, F = 1.452, *p* = 0.0606).

The area of remaining gray matter was measured in the same paradigm 8 weeks after SCI. The gray matter of spinal cords treated with EGCG was significantly more preserved compared to controls (1 mm, *p* = 0.025; 2 mm, *p* = 0.009; 4 mm, *p* = 0.029), compared to PP242 (2 mm, *p* = 0.002; 5 mm, *p* = 0.032) and EGCG + PP242-treated groups (2 mm, *p* = 0.01; 5 mm, *p* = 0.004). The combinatory treatment also significantly preserved gray matter more when compared to control (−2 mm, *p* = 0.018) (Two-way RM ANOVA F = 1.727, *p* = 0.0111). 

#### 3.2.2. Axonal Sprouting

**Figure 4:**
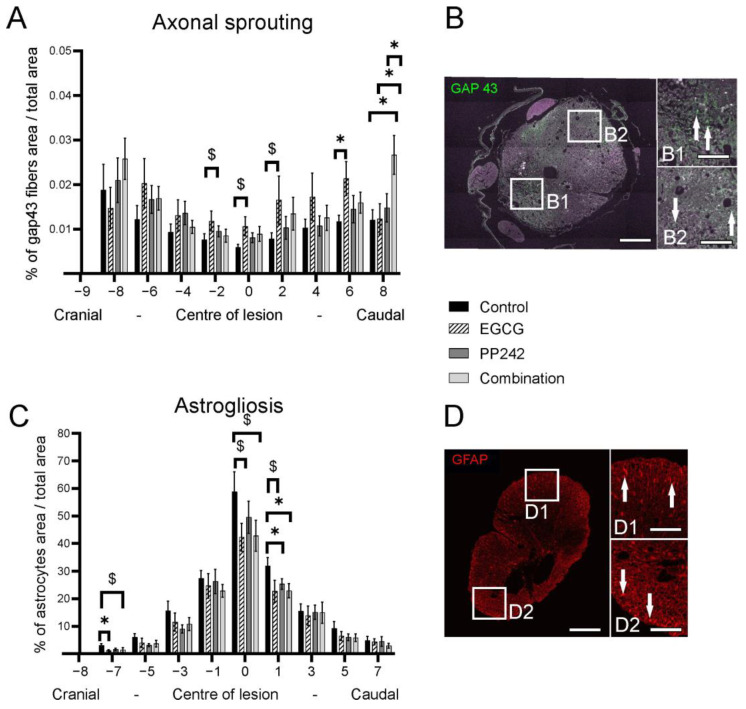
Histopathological and immunohistochemical investigation of the spinal cord (**A**,**B**). The axonal sprouting, as detected by GAP-43 expression, was significantly higher 6 mm and 8 mm caudally to the lesion center in the EGCG-treated group and EGCG + PP242-treated group compared to controls (**C**,**D**). In rats treated with EGCG, PP242, and EGCG + PP242, the GFAP-positive area was significantly reduced compared to the control, especially at the lesion site and in the immediate caudal region (* *p* < 0.05), ($ *p* 0.05–0.1) Scale bar 500 μm (**B**,**D**), 100 μm (B1, B2, D1, D2).

The GAP-43-positive area was measured to evaluate the level of axonal sprouting 8 weeks after SCI (Figure 4). The area was measured at every second millimeter of the spinal cord lesion at 8 mm cranially and caudally from the lesion center. Results were expressed as the ratio of GAP-43-positive area to the total spinal cord area. The EGCG-treated group had significantly higher axonal sprouting 6 mm caudal to the center of the lesion compared with the control group (6 mm, *p* = 0.036). At 8 mm caudal to the center of the lesion, the combined treatment caused higher axonal sprouting compared with controls, and also the PP242 and EGCG groups. (8 mm, *p* = 0.024; *p* = 0.043; *p* = 0.032, respectively) (Two-way RM ANOVA, F = 1.228, *p* = 0.2158) Figure 4A.

#### 3.2.3. Astrogliosis

The GFAP-positive area was measured to evaluate astrogliosis 8 weeks after SCI. The sections were collected from every second millimeter 7 mm caudally and 7 mm cranially from the lesion center. The results were expressed as the ratio of GFAP-positive area to the total spinal cord area. The EGCG, PP242, and EGCG + PP242 treatments reduced the GFAP-positive area (−7 mm, *p* = 0.027; 1 mm, *p* = 0.043; 1 mm, and *p* = 0.037, respectively). Figure 4B (Two-way RM ANOVA F = 0.7626, *p* = 0.7826).

#### 3.2.4. Phospho-pS6 Staining

**Figure 5:**
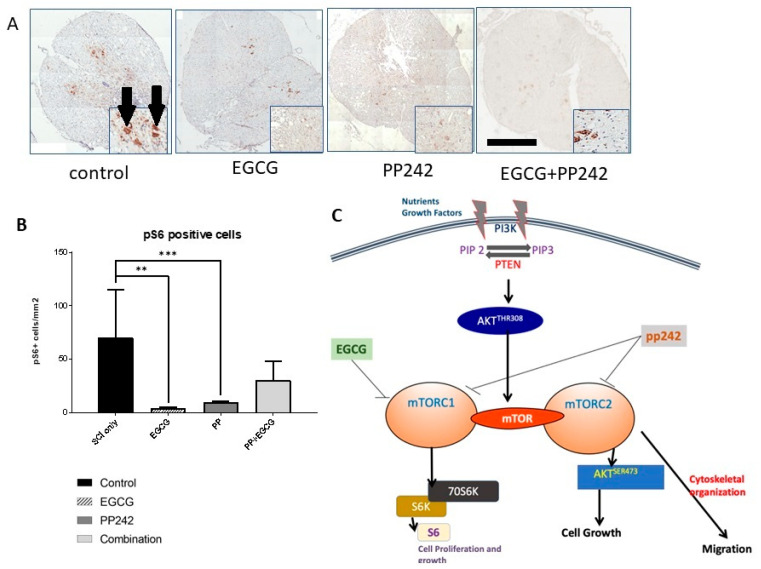
Phospho-S6-positive cells in spinal cord section at the lesion site (**A**). The photomicrograph shows staining of phospho-S6-positive (pS6) cells per mm^2^ in the spinal cord section from the lesion center site. Scale bar 500 μm (**B**) Quantitative analysis of pS6-positive cells showed a reduced number of pS6-positive cells in the lesion center following the treatment with EGCG (** *p* < 0.01) and PP242 (*** *p* < 0.001), and a trend towards significance was seen following treatment with EGCG + PP242 ($ *p* 0.05–0.1). The arrows show pS6-positive cells in the spinal cord gray matter. (**C**) Schematic representation of the mTOR pathway and its inhibition by EGCG and PP242 after spinal cord injury (SCI). Nutrient sensitive mTORC1 is activated by the canonical pathway that leads to activation of AKT, via conversion of phosphatidylinositol 4,5-bisphosphate (PIP2) to phosphatidylinositol (3,4,5)-trisphosphate (PIP3) by phosphoinositide 3-kinase (PI3K). Inhibition of mTORC1 and mTORC2 by pp242 leads to inhibition of the ribosomal protein S6 kinase 1 (S6K1), resulting in inhibition of the mTOR pathway. Abbreviations: mTOR, mechanistic target of rapamycin complex; mTORC1, mechanistic target of rapamycin complex 1; mTORC2, mechanistic target of rapamycin complex 2; PI3K, phosphoinositide 3-kinase; PIP2, phosphatidylinositol 4,5-bisphosphate2; PIP3, phosphatidylinositol (3,4,5)-trisphosphate; S6K, ribosomal protein S6 kinase; S6, ribosomal protein; Phosphatase and tensin homolog (PTEN).

Phospho-S6 staining was used to confirm the role of the mTOR pathway in SCI. Ribosomal protein S6 (pS6) is one of the major downstream targets and effectors of the mTOR signaling pathway. The number of pS6-positive cells was counted in the spinal cord lesion center and expressed as the number of pS6-positive cells per mm^2^. The number of pS6-positive cells was noticeably higher in control rats, whereas it decreased significantly following treatment with PP242 (*t*-test, *p* < 0.0001) or EGCG (*t*-test, *p* = 0.0002) compared with controls (Figure 5). Combined treatment with EGCG and PP242 also showed a reduction in the expression of pS6-positive cells, but only with a tendency to statistic significance.

#### 3.2.5. Expression of Intrinsic Genes

**Figure 6:**
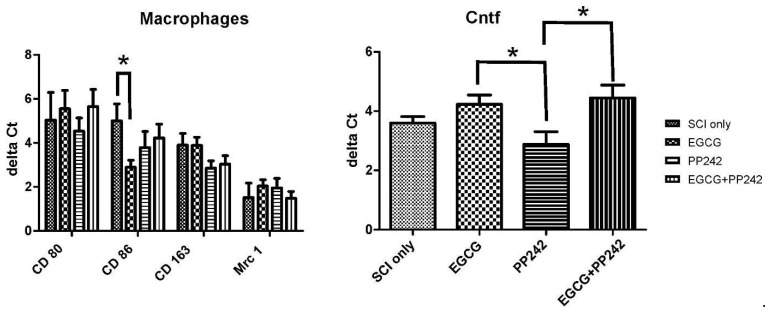
The qPCR analysis displayed lower expression of M1 macrophage marker, CD 86, after EGCG treatment compared to controls. The expression of Cntf (Ciliary neurotrophic factor) was significantly higher in the EGCG or PP242+EGCG treatment groups compared to PP242 or controls. This is evident from the higher expression of Cntf in EGCG-treated rats compared to the PP242-only group. * *p* < 0.05 indicates statistical significance.

The expression of genes related to M1 and M2 macrophages (CD80, CD86, CD 163, and Mrc 1) by qRT-PCR 8 weeks after the SCI was investigated. There was a significant difference in the expression of the marker for the M1 macrophages, as shown by reduced CD 86 levels after EGCG treatment (*t*-test, *p* = 0.0305). Expression of Cntf factor, a known stimulator of gene expression, cell survival, and differentiation in a variety of neuronal cells, was examined, and its expression was significantly higher after EGCG and EGCG + PP242 treatments compared to PP242 treatment alone (*t*-test, *p* = 0.03; 0.0396, respectively). Figure 6.

## 4. Discussion

The results of this study demonstrated that treatment with EGCG or PP242 significantly improved motor recovery. Combined treatment also showed improved sensitivity to mechanical stimuli; both effects were transient.

The green tea catechin, EGCG, possesses antioxidant activity with anti-inflammatory potential and neuroprotective characteristics [7,46]. EGCG is shown to increase α-secretase activity and hydrolysis of the TNF-α-converting enzyme to promote the cleavage of the α-c terminal fragment of APP in a model of Alzheimer’s disease [47,48]. Treatment with EGCG after SCI reduced the level of inflammatory cytokines, such as TNF-α and IL-1, and also suppressed levels of inducible nitric oxide synthase, cyclooxygenase-2, and myelin peroxidase. Furthermore, EGCG administration caused an anti-apoptotic response through suppression of Bax gene expression, as well as protein expression and enhancement of Bcl2, Bdnf, and Gdnf gene expression [28,49]. In addition, administration of EGCG following neural damage reduced neuropathic pain [9,10]. It has been shown that EGCG reduced edema and enhanced tissue protection and functional recovery after SCI [24,28,29]. Importantly, EGCG decreases the nuclear translocation of subunit p65 (RelA) of the NF-κB dimer, and therefore attenuates the canonical NF-κB pathway [8]. EGCG treatment led to higher gray matter preservation and caused higher axonal sprouting, as shown by increased expression of GAP-43 (Figure 4). These observations corroborate with our previous investigation that showed a reduced number of protoplasmic astrocytes in the spinal cord lesion following EGCG treatment [8]. Gene expression of Cntf factor was upregulated after EGCG treatment compared to PP242 treatment (Figure 6). The higher gene expression of Cntf remained significant even with the combined treatment. These results suggest that increased neuroregeneration via Cntf may have occurred through Cntf after SCI. Cntf is present exclusively in the nervous system where it can promote neurogenesis [50].

Gene expression of M1 and M2 macrophage polarization was examined, and CD 86 gene expression was reduced after EGCG treatment. This result is consistent with our previous findings that EGCG significantly modulated the inflammatory response after SCI by modulating inflammatory cytokines and macrophage polarization [8]. EGCG and PP242 are mTOR inhibitors. Both treatments significantly reduced phosphorylation of the S6 compared to the vehicle-treated group 7 days after SCI. The PI3K/Akt/mTOR pathway is involved in many physiological mechanisms, such as transcription, translation, cytoskeletal organization, and autophagy. PP242 is a new generation inhibitor of both the mTORC1 and mTORC2 complexes. Compared to PP242, EGCG has a wider range of other beneficial activities that enhance regeneration, such as antioxidative and anti-inflammatory activities. On the other hand, PP242 is considered to be neuroprotective, mainly due to its mTOR pathway inhibition. It has been shown in our previous research that EGCG has a protective effect on the spinal cord lesion development and functionality after injury. It has been shown that early application of EGCG following SCI led to better functional improvement compared to controls in the motor abilities of the rats. We found larger gray matter sparing and higher axonal sprouting compared to controls, caused by the suppression of NF-κB activity, upregulation of the expression of VEGF and FGF2, modulation of the expression of different macrophage markers, and cytokine production [8]. Our recent findings confirm that EGCG has a protective effect on gray matter following SCI due to the higher expression of genes for Cntf factor and mTOR pathway inhibition. In this study, we show that complex behavior, such as ladder walking and thermal sensitivity, is significantly improved after EGCG treatment (Figure 1 and Figure 2).

Autophagy and Akt signaling were evaluated in several prior studies. In a rat SCI model created by spinal cord compression at the level of T8, intraperitoneal administration at the time of SCI of 1 mg/kg Rapamycin or intraperitoneal administration of 3-MA 20 min prior to SCI was performed [2]. Immunohistochemistry has revealed increased Beclin1 along with western blot assay findings of decreased phosphorylated mTOR in the rapamycin group, suggesting a reduction in autophagy via mTOR inhibition. Apoptosis in neural tissue was decreased, as demonstrated by increased Bcl-2 and reduced Bax. Rapamycin that was given 1 mg/kg in another study, 4 h after SCI, inhibited the mTOR and significantly improved the Basso Mouse Scale (BMS). Improvement in the survival and decrease in neural destruction was seen by an increase in NeuN-positive cells and a decrease in TUNNEL-positive cells, respectively [51]. An inverse relationship between apoptosis and autophagy was demonstrated in a rat model of SCI where rapamycin was given in combination with 3-MA, an autophagy inhibitor. Functional use of BBB revealed that the rapamycin group had significantly higher scores, while 3-MA showed significantly lower scores [52]. The immune response and autophagy were evaluated in a rat model of SCI developed through balloon compression at the T8 level. mTORC1 inhibitor, rapamycin (5 mg/kg), and mTORC2 inhibitor, PP242 (5 mg/kg), inhibited the mTOR pathway and increased autophagy, as detected through an increase in LC3b-I expression. Furthermore, immune response was affected after rapamycin or PP242 treatment in the form of significantly lower levels of IL-1B MIP-1α compared to controls. These changes were reflected in improved BBB outcome at 7 days following SCI in both rapamycin and PP242 groups [52]. The role of the TOR pathway is consistent with other studies, in which rapamycin treatment prior to SCI showed significantly lower p-mTOR expression levels compared to the control. However, electroacupuncture increased p-mTOR and showed favorable results in imaging, histology, and functional recovery. This study therefore describes the association between mTOR pathway activity induced by electroacupuncture and the improved outcome after SCI in a rat model [53]. Alternatively, mTOR inhibition in the early phase after SCI has shown a reduction in neural stem progenitor cell (NSPC) numbers and a decrease in functional recovery [54].

In conclusion, the results of this study emphasize that treatment with EGCG and PP242 significantly improves recovery from SCI. EGCG is superior in improving locomotor performance, balance, and sensory functions. Thus, for the first time, it was demonstrated that EGCG improves recovery from SCI through modulation of the mTOR pathway, as shown by its ability to suppress S6 and significantly improve functional recovery. Despite this observation, the superiority of EGCG over PP242 could be caused not only due to the mTOR pathway modulation but also the other actions of EGCG, such as antioxidant activities. EGCG regulates signal transduction pathways, DNA methylation, mitochondrial function, and autophagy [55]. As EGCG has been used in many clinical studies [56,57,58,59,60], and since it is generally considered as safe, the transfer to clinical practice can be fast. PP242 has been confirmed in many preclinical investigations as an mTOR1 and mTOR2 pathway inhibitor and can activate autophagy [61]. The role of PP242 in the central nervous tissue must be further investigated to understand the specific mechanisms of how PP242 can protect neural tissue after injury.

## Data Availability

The data is contained within the article.
